# Bacterial microbiota associated with *Rhipicephalus sanguineus* (*s.l.*) ticks from France, Senegal and Arizona

**DOI:** 10.1186/s13071-017-2352-9

**Published:** 2017-09-07

**Authors:** Magalie René-Martellet, Guillaume Minard, Raphael Massot, Van Tran Van, Claire Valiente Moro, Luc Chabanne, Patrick Mavingui

**Affiliations:** 1 VetAgro Sup campus vétérinaire de Lyon, Université de Lyon, Marcy l’Etoile, France; 2 UMR 0346 EPIA, INRA-VetAgro Sup, Saint Genès Champanelle, Marcy l’Etoile, France; 3 Université de Lyon, Lyon, France Université Lyon 1, Villeurbanne, France; CNRS, UMR 5557, Ecologie Microbienne, Villeurbanne, France; INRA, UMR1418, Villeurbanne, France; 4 Metapopulation Research Centre, Department of Biosciences, University of Helsinki, Helsinki, Finland; 5 Université de La Réunion, CNRS UMR 9192, INSERM U1187, IRD UMR 249, Unité Mixte Processus Infectieux en Milieu Insulaire Tropical (PIMIT), Plateforme de Recherche CYROI, Saint-Denis, La Réunion France

**Keywords:** *Rhipicephalus sanguineus*, Symbionts, *Coxiella*, *Rickettsia*, *Bacillus*

## Abstract

**Background:**

Ticks of the group *Rhipicephalus sanguineus* (*sensu lato*) are distributed worldwide and are major pathogen vectors of both dogs and humans. Previous phylogenetic reconstructions have suggested the existence of two main lineages within this group, “Tropical” and “Temperate”. Symbiotic interactions contribute to vector development, survival, reproduction and competence. The diversity of microbial communities associated with different populations of *R. sanguineus* (*s.l.*) remains poorly characterized, however, this knowledge will aid in future studies of hosts-microbiota-pathogen interactions. To gain insight into the bacterial communities associated with *R. sanguineus* (*s.l.*) ticks, 40 specimens from France, Senegal and Arizona were analyzed by high-throughput *16S* amplicon sequencing. All tick specimens were taxonomically classified using the mitochondrial *12S* rDNA gene, which provides sufficient phylogenetic resolution to discriminate different lineages of *R. sanguineus*.

**Results:**

*Rhipicephalus sanguineus* (*s.l.*) samples from Senegal belonged to the “Tropical” lineage, samples from France belonged to the “Temperate” lineage, whereas both lineages were identified in samples from Arizona. Regardless of origin, each bacterial microbiota was dominated by three genera: *Coxiella*, *Rickettsia* and *Bacillus*. *Rickettsia* and *Coxiella* were the two main genera found in females whereas males had a higher proportion of *Bacillus*. Significant differences of relative abundances were evidenced between specimens from different geographical origins.

**Conclusions:**

This study highlights differences in the microbiota composition within *R. sanguineus* (*s.l.*) specimens from different genotypes, genders and geographical origins. This knowledge will help in future studies of the symbiotic interactions, biology and vector competence of the *R. sanguineus* (*s.l.*) complex.

**Electronic supplementary material:**

The online version of this article (10.1186/s13071-017-2352-9) contains supplementary material, which is available to authorized users.

## Background

Infectious diseases are the second most common cause of death worldwide, behind cardiovascular diseases. Many arthropod vectors are known to transmit infection of “vector-borne diseases”. The transmission cycles of such diseases rely on complex pathosystems in which vectors, hosts and microorganisms (from pathogens to mutualists) interact within changing ecosystems. This complexity, and the diversity of players/partners involved in pathosystems, can make vector control difficult in the field. It has been demonstrated that some microbe-microbe interactions that occur within vectors, in particular mosquitoes, can interfere with life-history traits, including vector competence [1–6]. Consequently, increasing numbers of studies of vector-borne pathogen transmission and control now adopt integrative approaches that take all interacting players of each pathosystem into account.

Globally, ticks are considered as the second most important disease vectors after mosquitoes [7]. But in Europe, ticks are considered the most common vector of both human and veterinary diseases to date [8]. The last decades have witnessed the emergence of new tick-borne diseases and changes in the geographical distribution of previously known tick-borne pathogens. This suggests that the emergence and re-emergence of several tick-borne pathogens is likely to be of significant socioeconomic burden in the future [8–10].

*Rhipicephalus sanguineus* was first described by Latreille in 1806 using specimens collected from Gallia [11]. It is one of the most prevalent ticks found on dogs in southern France [12] and specimens attributed to the species have been described nearly worldwide. The taxonomic status of *R. sanguineus* remains controversial and has been subject to numerous debates over the last decades [11, 13–18]. The main conclusions of these studies referred to the absence of a reliable pictorial key to morphologically identify *R. sanguineus* (Latreille, 1806), the existence of different populations within the morphotype *R. sanguineus* and the possible confusion with *R. turanicus.* Therefore, all the specimens harboring the original pictorial keys annotated in the first description were gathered under the taxon *R. sanguineus* (*sensu* *lato*) (*s.l.*). Within *R. sanguineus* (*s.l.*), two parapatric lineages were identified based on phylogeny of mitochondrial *16S* and *12S* rDNA genes: a temperate southern American/western European lineage and a tropical southern American/African lineage [13, 17, 19].

*Rhipicephalus sanguineus* (*s.l.*) is highly specialized for dogs and, thus, occasionally colonizes human habitats. It is considered as a significant vector due to its ability to replicate and transmit many bacterial and parasitic agents, including *Ehrlichia canis*, *Mycoplasma haemocanis*, *Babesia vogeli*, *Hepatozoon canis* to carnivores and *Rickettsia conorii*, the agent of the Mediterranean spotted fever, to humans [18, 20]. Because of its vector role, as well as its ability to colonize human habitats, this species is a real threat to human and animal health [21].

Metazoans interact with a broad community of microorganisms [22]. Those interactions are called symbiosis and can result in positive, negative or neutral effects on both partners [23, 24]. Symbiont-based strategies have been proposed as a tool for vector control in mosquitoes [3] as well as in ticks [25]. Such approaches will gain in efficiency if fundamental knowledge is acquired on host-microbiota-pathogens interactions and dynamics. One of the first steps to apply such strategies is the description of the microbial communities that interact with populations of *R. sanguineus* (*s.l.*) *in natura*. To date, few studies have aimed at identifying bacterial communities within *R. sanguineus* (*s.l.*). Analysis of specimens from laboratory colonies [26] or collected from the same area have shown the dominance of the genera *Coxiella* and *Rickettsia*, as well as the detection of the intracellular bacteria *Wolbachia* spp. [27, 28]. In particular, a multicentric study conducted in southern France from 2010 to 2012 showed significant regional differences in the prevalence of *R. sanguineus* (*s.l.*) ticks infection by *Babesia vogeli*, a hemoprotozoan pathogen of dogs [12]. As no genetic difference (using *16S* and *12S* rDNA fragments) was shown between tick specimens collected in the sampled regions, the possibility that such differences in prevalence of infection could be associated with differences in microbiota within *R. sanguineus* (*s.l.*) specimens was suggested [12].

To get further insight into bacterial communities hosted by *R. sanguineus* (*s.l.*) ticks, specimens were collected in four areas of southern France, one area in Arizona and one area in Senegal. To overcome misinterpretations linked to the controversial taxonomic status, all *R. sanguineus* (*s.l.*) specimens used were genetically characterized by sequencing of a 400 bp mitochondrial *12S* rRNA fragment. Then, high-throughput *16S* rRNA amplicon sequencing was performed to describe the diversity and structure of bacterial communities interacting with *R. sanguineus* (*s.l.*) from different origins.

## Methods

### Sampling

The survey was conducted in four areas from southern France (Corsica, Drôme, Gard and Var), one location in Senegal (Dakar area) and one location in USA (San Carlos area, Arizona) (Table 1). All study sites were selected because of the high prevalence of *R. sanguineus* (*s.l.*) ticks recorded from previous surveys. Sampling in Senegal and Arizona was performed in order to compare their bacterial microbiota to that of the French populations, encompassing the two lineages known as “Temperate” and “Tropical”. The four French sites also allowed exploration of possible differences in bacterial communities of ticks from different locations within the same country. Ticks were collected from either infested dogs or the environment by visual picking, flagging or CO_2_ trapping. Adults and nymphs were collected at each site. All ticks were stored in 70% ethanol until used. Collected ticks were sorted by developmental stage (nymphs, adult males and females) and were identified under light microscopy using pictorial keys [29, 30], allowing selection of *R. sanguineus* (*s.l.*). Then ticks were randomly selected within each group to be as representative for each stage and location as possible. Taking into account the complex taxonomic status of *R. sanguineus* (*s.l.*), and the possible misidentification with other species of the genus, all morphological identification was confirmed by amplification and sequencing of a 400 bp fragment of mitochondrial *12S* rRNA gene. In the case of ticks collected from dogs, only the least blood-engorged ticks were kept (Additional file 1: Table S1).

**Table 1:** Information on the 40 *R. sanguineus* (*s.l.*) specimens used for analyses of bacterial diversity

Sample	Location (coordinates)	Sex/Stage	Collection method	Haplotype
AR-1	San Carlos, Arizona (33°20′N, 110°27′W)	F	From the environment^a^	5
AR-2		F		5
AR-3		M		5
AR-4		M		6
AR-5		M		5
FR-CO1	Bastia, Corsica, France (42°41′N, 9°27′E)	M	From dogs	1
FR-CO2		F		1
FR-CO3		M		1
FR-CO4		N		1
FR-CO7		F		1
FR-D1	La Bégude de Mazenc, Drôme, France (44°32′N, 4°56′E)	F	From dogs	1
FR-D2		F		1
FR-D3		F		1
FR-D4		F		1
FR-D5		F		1
FR-D6		F		1
FR-D7		M		1
FR-G1	Sommières, Gard, France, (43°47′N, 4°05′E)	F	From dogs	1
FR-G2		F		1
FR-G3		F		1
FR-G4		F		3
FR-G6		N		1
FR-G7		N		1
FR-G13		F	From the environment^b^	3
FR-G14		F		1
FR-G5	Aigues-Vives, Gard, France (43°42′N, 4°13′E)	F	From a dog	1
FR-G11		F	From the environment^c^	3
FR-G9	Saint-Gilles, Gard, France (43°40′N, 4°26′E)	M	From dogs	1
FR-V4	Toulon, Var, France (43°07′N, 5°55′E)	F	From dogs	3
FR-V5		F		3
FR-V6		F		1
FR-V7		M		1
FR-V8		M		1
SEN-1	Dakar, Senegal (14°43′N, 17°25′W)	F	From dogs^d^	7
SEN-3		F		7
SEN-4		F		8
SEN-5		F		7
SEN-6		M		6
SEN-7		M		8
SEN-8		F		7

*Abbreviations*: F, female; M, male; N, nymph

^a^Using CO_2_ traps in sub-urban private houses

^b^Using the flagging method, in a rural location, along a small wooded river occasionally frequented by dogs

^c^Using the flagging method, along the river “Le Vidourle” in a park within this middle town of southern France

^d^Dogs from the same kennel

### DNA extraction

DNA was extracted as previously described [31] and the quality assessed by PCR amplification of a 400 bp fragment of mitochondrial *12S* rRNA gene using primers targeting ticks [19]. Quantification of total DNA was systematically performed after each DNA extraction using a spectrophotometer (SAFAS, Monaco, Principality of Monaco). For each series of extractions, a negative control corresponding to an extraction tube without a tick sample was performed in parallel.

#### *Rhipicephalus sanguineus* (*s.l.*) haplotyping

Products of PCR amplification of the mitochondrial *12S* rRNA genes of all *R. sanguineus* (*s.l.*) ticks used in the study were sequenced by BIOFIDAL-DTAMB (FR BioEnvironment and Health, Lyon, France). Sequences obtained were manually corrected by visual analysis of the electropherogram, aligned and assembled by haplotypes using Bioedit v7.0.5.3 [32]. The consensus sequence of each *R. sanguineus* (*s.l.*) haplotype was then used as query sequences using BLAST against the NCBI nucleotide database. The 400 bp sequences of *R. sanguineus* (*s.l.*) ticks were then aligned with sequences of *R. sanguineus* (*s.l.*) from different parts of the world and a phylogenetic tree was built using the Maximum Likelihood method. The nucleotide evolution model of Hasegawa, Kishino and Yano was selected based on the Akaike Information Criterion corrected following the previously described method [33]. The mitochondrial *12S* rDNA sequences from ticks of *R. sanguineus* (*s.l.*) used in the study were deposited in the GenBank database under the accession numbers KU255848–KU255856.

### Bacterial *16S* rDNA analysis

Amplification of V5-V6 rrs hypervariable regions was performed in triplicates as previously described [34]. Briefly, 30 ng of DNA were amplified with 200 nM of each primer, 1.75 U of Expand High Fidelity Enzyme Mix (Roche, Basel, Switzerland), 1× Expand High Fidelity Buffer (Roche, Basel, Switzerland), 0.06 mg/ml of T4 gene 32 protein (New England Biolabs, Evry, France), 0.06 mg/ml of bovine serum albumin (New England Biolabs, Evry, France), and 40 μM of dNTP. The 784F (5′-AGG ATT AGA TAC CCT GGT A-3′) and 1061R (5′-CRR CAC GAG CTG ACG AC-3′) primers used also contained an 8 bp multiplex barcode and Illumina adapters. Amplifications were performed on Biorad C1000 thermal cycler (Biorad, CA, USA), with 5 min at 95 °C, 35 cycles with 40 s of denaturation at 95 °C, 1 min of hybridization at 54.2 °C, 30 s of extension at 72 °C and a final extension step of 7 min at 72 °C. Amplicons were purified with Agencourt AMPure XP PCR Purification kit (Beckman Coulter, Villepinte, France) and quantified using the Quant-iT Picogreen dsDNA Assay Kit (Life Technologies, NY, USA). Then an equimolar mix of each amplicon was prepared for sequencing. A negative control (pool of 3 amplifications of a blank extraction) was sequenced with the library pool. Sequencing of libraries was performed on the Illumina Miseq Platform (2 × 250-bp pared-end reads) by ProfileXpert - ViroScan 3D (Lyon, France). Analysis of the V5-V6 rrs sequences was performed with Mothur v.1.33.3 pipeline [35]. Briefly, sequences were trimmed based on (i) index presence with less than two errors on primers; (ii) size comprised between 250 bp and 350 bp; and (iii) no ambiguous sequences and less than 9 homopolymers. Sequences were then aligned on Silva v.119 and chimeras were removed with UCHIME implemented in Mothur [36]. A total of 8,972,702 sequences of good quality were available for the analysis. According to neighbor-joining method, similar sequences were then clustered as a unique OTU if they harbored less than 3% divergence. The taxonomy assignments of OTUs were performed with Naive Bayes Classifier using Silva database v.119. Contaminants were removed from the analysis using a homemade script that suppresses OTUs if their relative abundance (proportion of reads) in a given sample was not at least 10× higher than in the negative control. For all the analyses, an even number of sequences were used. Richness (sobs, chao1), α-diversity (1/λ, H′) and β-diversity (Bray-Curtis) indices were calculated (Additional file 1: Table S2). Non-parametric analysis of microbiota homogeneity (Homogeneity of Variance, HOMOVA) and differentiation (Analysis of Molecular Variance, AMOVA) among samples were performed with MOTHUR pipeline, following the method previously described [37]. The HOMOVA analysis compares the homogeneity of the communities within the groups and the AMOVA compares the differences of the microbial communities among the groups. Fastq files were deposited on the European Nucleotide Archive under the project accession number (PRJEB21785).

## Results

### Haplotyping of *R. sanguineus* (*s.l.*) ticks and samples selection

Forty-nine *R. sanguineus* (*s.l.*) ticks were first selected according to morphological criteria and named: AR-1–AR-5; FR-CO1–FR-CO7; FR-D1–FR-D7; FR-G1–FR-G14; FR-V1–FR-V8; SEN-1–SEN-8, according to their geographical origin (AR: Arizona; FR-CO: France-Corsica; FR-D: France-Drôme; FR-G: France-Gard; FR-V: France-Var; SEN: Senegal). Among these, 49 individuals, analyses of the 400 bp segment of mitochondrial *12S* rRNA gene distinguished 8 different haplotypes (haplotypes 1–8). After BLAST analysis, ticks of haplotypes 1 (KU255848), 2 (KU255849), 3 (KU255850), 4 (KU255851) and 5 (KU255852), all collected in France or Arizona, showed 99–100% homology with *R. sanguineus* (*s.l.*) ticks sequences from western Europe, USA and southern South America. Haplotypes 6 (KU255853 and KU255854), 7 (KU255855) and 8 (KU255856), were associated with ticks collected in Senegal, except one sample (AR-4) that was collected in Arizona. Those sequences showed 98–99% of homology with *R. sanguineus* (*s.l.*) ticks sequences from Brazil (AY559842) and Asia (JX416325, DQ003001 and AY987377). The proportion of nucleotide identity between the 8 *R. sanguineus* (*s.l.*) haplotype sequences identified in the study is presented in Table 2.

**Table 2:** Sequence identity matrix between the eight *R. sanguineus* (*s.l.*) haplotypes detected in the study

	haplotype 1^a^	haplotype 2^a^	haplotype 3^a^	haplotype 4^a^	haplotype 5^a^	haplotype 6^b^	haplotype 7^b^	haplotype 8^b^
haplotype 1	–							
haplotype 2	0.982	–						
haplotype 3	0.997	0.979	–					
haplotype 4	0.984	0.997	0.982	–				
haplotype 5	0.994	0.977	0.997	0.979	–			
haplotype 6	0.745	0.742	0.745	0.745	0.742	–		
haplotype 7	0.747	0.745	0.747	0.747	0.745	0.997	–	
haplotype 8	0.750	0.747	0.75	0.75	0.747	0.995	0.997	–

^a^ *R. sanguineus* (*s.l.*) haplotypes of the “Temperate” lineage

^b^ *R. sanguineus* (*s.l.*) haplotypes of the “Tropical” lineage

**Fig. 1:**
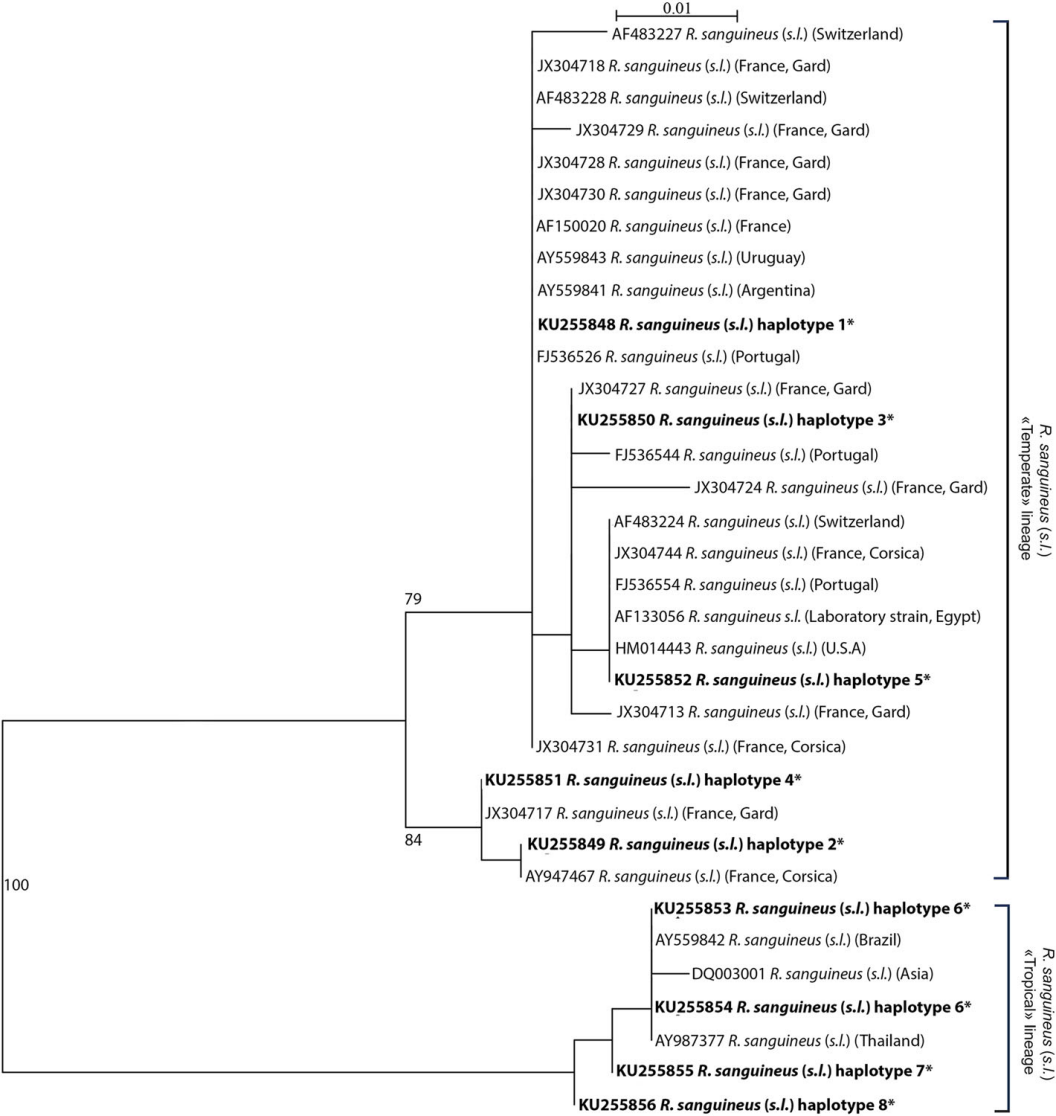
Phylogenetic analysis of *R. sanguineus* (*s.l.*) ticks based on mitochondrial *12S* rRNA gene. Sequences (400 bp) of *R. sanguineus* (*s.l.*) ticks from France, Senegal and Arizona were assembled by haplotypes and compared to sequences of *R. sanguineus* (*s.l.*) from different parts of the world. Identification and GenBank accession numbers are indicated for each sample. Countries or regions where individuals were isolated are also given in brackets. The Maximum Likelihood phylogenetic tree was constructed using the Hasegawa, Kishino and Yano method with bootstrap analysis of 1000 pseudoreplicates. Numbers on branches indicate support for each clade ≥ 75%. Subsequent analyses using Kimura’s two-parameter (K2P) distance and parsimony methods in the same conditions confirmed the topology of the tree (not shown)

Phylogenetic analysis (Fig. 1) confirmed the existence of two monophyletic clusters. The first cluster, labeled as the “Tropical” lineage, included *R. sanguineus* (*s.l.*) specimens from Asia and the northern part of South-America (Brazil). The second cluster, labelled as the “Temperate” lineage, included sequences from Europe, Egypt, USA and southern South America (Argentina, Uruguay). All *R. sanguineus* (*s.l.*) sequences of the study originating from Senegal clustered in the “Tropical” lineage, all *R. sanguineus* (*s.l.*) sequences from France clustered in the “Temperate” lineage whereas sequences of *R. sanguineus* (*s.l.*) from Arizona were distributed within both lineages. Interestingly, within the “Temperate” cluster, several specimens of *R. sanguineus* (*s.l.*) from France, including specimens of the study assigned to haplotypes 2 and 4, formed a monophyletic sub-group supported by a bootstrap value of 84%.

Finally, among the 49 samples initially selected, two samples assigned to haplotypes 2 and 4 (FR-CO6 and FR-G8) were excluded from the study because of their genetic divergence within the “Temperate” lineage. Among the remaining ticks, five were discarded for insufficient amplification of the V5-V6 rrs hypervariable regions (samples FR-V1, FR-V2, FR-V3, FR-G10 and FR-G12) and 2 were excluded due to insufficient read number after Miseq sequencing (samples SEN-2 and FR-CO5). A total of 40 samples were kept for subsequent analyses of bacterial diversity. Information on the 40 analyzed samples is given in Table 1.

### Bacterial diversity

**Fig. 2:**
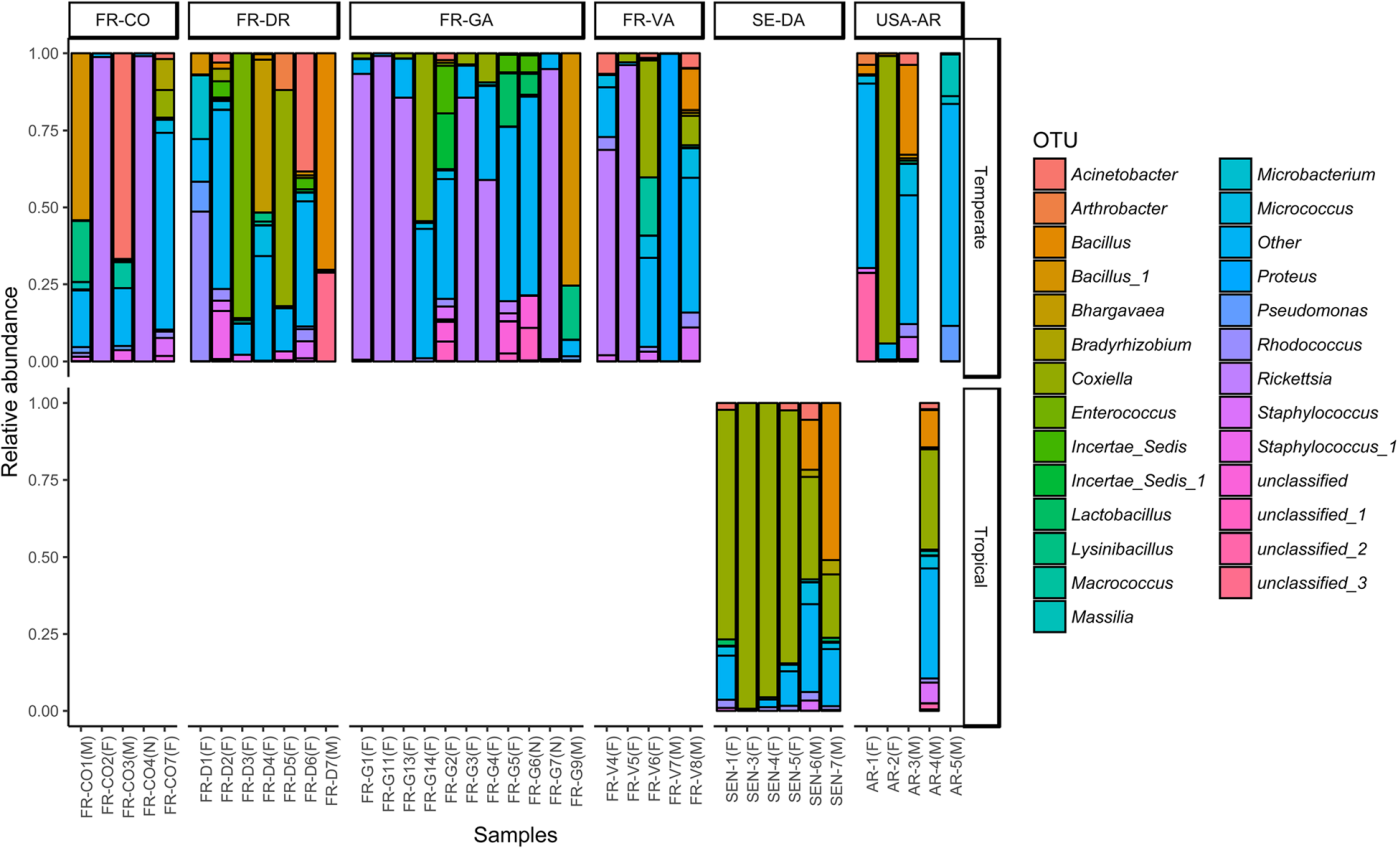
Histograms of bacterial Operational Taxonomic Units (OTU) abundances. The OTUs relative abundances are represented for samples collected in France-Corse (FR-CO), France-Drome (FR-DR), France-Gard (FR-GA), France-Var (FR-VA), Senegal-Dakar (SE-DA), USA-Arizona (USA-AR) as well as for “Temperate” or “Tropical” lineage. Samples of males (M), females (F) and nymphs (N) samples are also specified. OTUs which do not reach a relative abundance of 0.1 in at least one sample were pooled in a category named “Other”

The number of sequences obtained was between 85,388–386,478 per sample. This depth was sufficient to reach reliable diversity estimates (Coverage > 0.999) (Additional file 1: Table S2). The bacterial estimated richness of *R. sanguineus* (*s.l.*) was comprised of between 34 and 562 OTUs per individual (Chao1 = 243 ± 132) (Additional file 1: Table S2). The bacterial microbiota of *R. sanguineus* (*s.l.*) was dominated by 3 genera (Fig. 2), namely *Coxiella* (phylum *Proteobacteria*, class *Gammaproteobacteria*), *Rickettsia* (phylum *Proteobacteria*, class *Alphaproteobacteria*) and *Bacillus* (phylum *Firmicutes*, class *Bacilli*). Both *Coxiella* and *Rickettsia* genera represented up to 99% of the total *R. sanguineus* (*s.l.*) microbiota. However, some samples had significantly higher proportions of *Bacillus* (up to 75%).

**Fig. 3:**
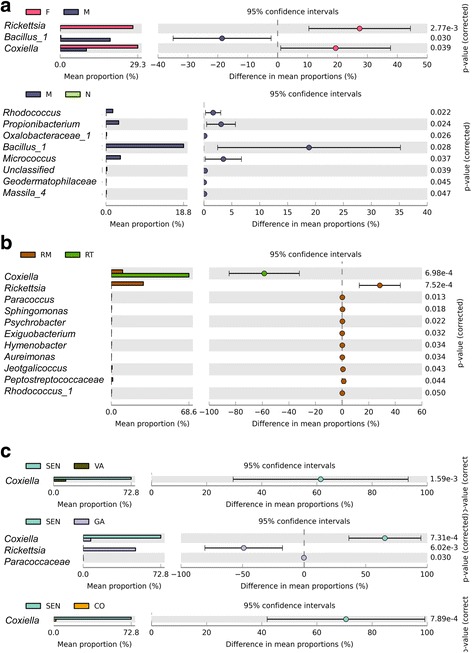
Extended error bar plots of the most abundant OTUs associated with the covariates. Differences of abundances among OTUs were tested with a Welsh corrected *t-*test for *R. sanguineus* (*s.l.*) from: **a** Nymphs (N), females (F) and males (M); **b** “Tropical” lineage (RT) and “Temperate” lineage (RM); **c** Senegal (SEN); France-Var (VAR), France-Gard (GA) and France-Corsica (CO). The extended error bars represent the 95% confidence interval of the fold change in relative abundance for an OTU between two modalities

The effect of sample categories (sex/stage), origin, habitat and their interactions on the β-diversity was estimated by a permutational multivariate analysis of variance (adonis ANOVA test) with 999 permutations. Those three factors were correlated with 48% of the overall variability. Only the sample categories, namely males, females, nymph (adonis permutational ANOVA: *F*_(2,28)_ = 2.29, *P* = 0.013) and the origin (adonis permutational ANOVA: *F*_(2,28)_ = 2.02, *P* = 0.002) showed a difference in the microbiota similarity. Any differences in heterogeneity were evidenced between sample categories. The females harbored a microbiota dominated by both genera, *Rickettsia* and *Coxiella*, whereas the males were mainly associated with a higher proportion of *Bacillus* (Welsh corrected t-test: *t* = -2.4, *df* = 10, *P* = 0.03) (Fig. 3a). In addition, adults harbored a higher proportion of *Rhodococcus*, *Propionibacterium*, *Micrococcus* and *Bacillus*, compared to nymphs (Fig. 3b). Significant differences in the microbiota homogeneity (higher within group similarity) and composition were observed between “Tropical” and “Temperate” tick lineages (HOMOVA: *Bv* = 2.19, *P* < 0.001; AMOVA: *Fs*_(1,37)_ = 4.46, *P* = 0.001) (Fig. 3b). However, the genetic divergence is confounded with the sampling site effect as the “Tropical” genotype was mainly associated with specimens collected from Senegal. Consequently, the differences between “Tropical” and Mediterranean populations reflected also the differences between Senegal and other locations. Indeed, Senegalese individuals harbored a more homogenous microbiota than those from France-Drôme and France-Gard (HOMOVA: *Bv* = 1.75, *P* = 0.002 and *Bv =* 1.19, *P* < 0.001, respectively). They also harbored a different microbiota from those collected in France-Corse, France-Gard and France-Var (AMOVA: *Fs*_(1,9)_ *=* 4.64, *P* = 0.002; *Fs*_(1,15)_ *=* 6.64, *P* < 0.001 and *Fs*_(1,9)_ *=* 3.53, *P* = 0.003, respectively). Generally, a higher abundance of *Coxiella* was associated with Senegalese specimens, compared to those of the last three populations from France (Fig. 3c).

Within specimens collected from France, microbiota of female ticks collected in Gard was characterized by a high abundance of *Rickettsia* and *Coxiella* compared to ticks from the other three sites. However, the number of samples is not sufficient to generalize those observations with a statistical analysis.

## Discussion

Few studies have aimed at identifying bacterial communities within ticks to date [25–28]. To provide new insight in bacterial communities hosted by *R. sanguineus* (*s.l.*) ticks, a survey of natural tick populations was conducted in 2014 in four locations at southern France, as well as in one site in Senegal and one site in Arizona. Taking into account the current controversial taxonomic status of *R. sanguineus* (*s.l.*) ticks, all specimens used in the study were genetically characterized by sequencing of a 400 bp mitochondrial *12S* rRNA fragment. As expected, all *R. sanguineus* (*s.l.*) sequences associated with Senegalese and French individuals were associated with the “Tropical” and “Temperate” lineages, respectively. In Arizona, ticks of both lineages were detected, which suggested that individuals from those two groups live in sympatry within this site. Two specimens were discarded from subsequent analyses because of their genetic divergence with the genotype of the most frequently encountered under “temperate” regions in the study. Analysis of bacterial communities within those particular specimens would result in difficulties in results interpretation.

High-throughput screening of variable V5-V6 bacterial *16S* rRNA gene was performed on all the forty selected *R. sanguineus* (*s.l.*) specimens. We found that the bacterial microbiota of *R. sanguineus* (*s.l.*) was dominated by three genera, namely *Coxiella*, *Rickettsia* and *Bacillus* representing up to 99% (*Coxiella* and *Rickettsia*) and 75% (*Bacillus*) of the total relative abundance. Interestingly, females harbored a microbiota dominated by *Rickettsia* and *Coxiella*, whereas males harbored a higher proportion of *Bacillus*. Previous surveys on both genders have also shown that *R. sanguineus* (*s.l.*) ticks were mainly infected by the symbiotic/pathogenic bacterial genera *Coxiella* and *Rickettsia* [27, 28, 38]. *Coxiella*-like endosymbionts have already been shown to be mainly associated with females [27]. They can colonize Malpighian tubules, ovaries of females and can also be maternally transmitted [27, 28]. If no clear tissue tropism was detected for *Rickettsia* sp. in *R. sanguineus* (*s.l.*), maternal transmission of such symbionts has previously been observed [28, 39–41]. Lalzar et al. also suggested that a competition for the ovary colonization occurs between *Coxiella* sp. and *Rickettsia* sp. [28]. Our results support such hypothesis, since individuals infected by *Rickettsia* spp. or *Coxiella* spp. bacteria were mainly females and were found dominantly infected by either one of those symbionts, but never both at the same abundance.

*Coxiella-*like endosymbionts are widespread among the hard ticks, in which they present patterns of co-evolution and genome reduction, which often occur in vertically-inherited endosymbionts [38, 42, 43]. Genome comparisons between *Coxiella-*like endosymbiont from the tick *Amblyomma americanum* and the Q fever ethiological agent *Coxiella burnetii* revealed an enrichment of genes involved in B vitamins and cofactors metabolism within the genome of the symbiotic strain [43]. The authors suggested that those bacterial functions are involved in a mutualistic interaction with the tick host by compensating nutritional deficiencies. Such a hypothesis is consistent with the observed decreases in ticks fecundity and life-history traits successive to the symbiont elimination [44]. The possible impact of *Coxiella* spp. and *Rickettsia* spp. on *R. sanguineus* (*s.l.*) survival, reproduction and vector competence needs to be explored further as it could open avenues for new control strategies.

Several species from the genus *Bacillus* were shown to colonize Arthropods. As an example, *Bacillus thuringiensis*, producing the Cry and Cyt toxins, has been broadly used for its biopesticide properties on Insects, whichhave shown interesting properties on Acarians [45, 46]. *Bacillus* spp. have previously been detected in several tick genera such as *Ixodes*, *Amblyomma*, *Aponomma*, *Haemaphysalis* and the species *Rhipicephalus* (*Boophilus*) microplus [47]. In the present study, *Bacillus* spp. represent 75% of the total relative abundance within *R. sanguineus* (*s.l.*) ticks and was mostly associated with males. The nature of the association between male *R. sanguineus* (*s.l.*) ticks and *Bacillus* spp. has not yet been characterized.

Finally, our results suggest variations of microbiota composition within *R. sanguineus* (*s.l.*) ticks related to origin. Similarly, *Ixodes* ticks originating from eastern USA presented a significant differentiation of their microbiota according to the distance among local populations [48]. In that study, variations in *Rickettsia* spp. and *Enterobacteriaceae* abundances were mainly responsible for those differentiations. In *R. sanguineus* (*s.l.*), site variations were related to a shift in the dominant taxa of *Coxiella* spp. to *Rickettsia* spp., and could be attributed to habitats or genetic changes associated with the ticks’ populations belonging to “Temperate” or “Tropical” lineages. Interestingly, *R. sanguineus* (*s.l.*) ticks from Gard are associated with a highest relative abundance of *Rickettsia* compared to other French sampling sites. The Gard region has also been identified as a hotspot for the prevalence of the hemoprotozoan *Babesia vogeli* in dogs [12]. During the development of the *Babesia* protozoa in the tick the following events occur: (i) sexual reproduction in the lumen of the digestive tract then (ii) invasion of different organs, including Malpighian tubules and the ovaries [49, 50]. Further investigations should be conducted to determine whether interactions between bacteria and the protozoa occur in ticks, and if they could interfere with its development or transmission as it has been previously shown with *Plasmodium falciparum* in mosquitoes [51].

Even though we have selected ticks that were not visually engorged, samples from some regions were collected on dogs and might have already ingested blood. Blood ingestion is responsible of transient changes in the microbial communities, and might have had an influence on our results [52]. More controlled studies should be conducted in order to tease apart those effects.

## Conclusions

This study used a high-throughput sequencing approach to characterize the bacterial microbiota associated with *R. sanguineus* (*s.l.*) ticks obtained from different origins. Our results highlight differences in the structure of the microbiota, according to tick genotypes, geographical origin of specimens and stages (nymphs, male or female). Three dominant bacterial genera, namely *Rickettsia*, *Coxiella* and *Bacillus*, emerged from these analyzes with strong correlations with the samples category, geographical origin and lineage. The genus *Coxiella* is strongly associated with “Tropical” ticks from Africa whereas the genus *Rickettsia* is mainly found in “Temperate” ticks from the Gard region of France. This study is the first comprehensive overview of the structure of the microbiota, and its variation factors, within *R. sanguineus* (*s.l.*) ticks. These results provide a basis for future work on symbiotic interactions, biology and vector competence within the *R. sanguineus* (*s.l.*) complex.

## Additional file

Additional file 1: Table S1. Samples used for the 16 s rDNA high throughput sequencing. **Table S2.** Alpha diversity indices. (DOCX 23 kb)

## Abbreviations

AMOVA: Analysis of molecular variance
ANOVA: Analysis of variance
HOMOVA: Homogeneity of molecular variance
OTU: Operational taxonomic units
*s.l.*: *sensu lato*
*s.s.*: *sensu stricto*
