# Supplementary material for: Bacterial microbiota associated with Rhipicephalus sanguineus (s.l.) ticks from France, Senegal and Arizona
Source: Parasit Vectors. 2017 Sep 7;10:416. doi: 10.1186/s13071-017-2352-9 (PMC5591579; doi:10.1186/s13071-017-2352-9)
Supplement: Additional file 1: Table S1. — Samples used for the 16 s rDNA high throughput sequencing. Table S2. Alpha diversity indices. (DOCX 23 kb) [file 13071_2017_2352_MOESM1_ESM.docx]

| samples | location (coordinates) | stade1 | collection method | haplotype | Clade |
| --- | --- | --- | --- | --- | --- |
| AR-1 | USA (Arizona) | F | Environment | 5 | Clade1 |
| AR-2 | USA (Arizona) | F | Environment | 5 | Clade1 |
| AR-3 | USA (Arizona) | M | Environment | 5 | Clade1 |
| AR-4 | USA (Arizona) | M | Environment | 6 | Clade2 |
| AR-5 | USA (Arizona) | M | Environment | 5 | Clade1 |
| FR-CO1 | France (Corsica) | M | Dogs | 1 | Clade1 |
| FR-CO2 | France (Corsica) | F | Dogs | 1 | Clade1 |
| FR-CO3 | France (Corsica) | M | Dogs | 1 | Clade1 |
| FR-CO4 | France (Corsica) | N | Dogs | 1 | Clade1 |
| FR-CO7 | France (Corsica) | F | Dogs | 1 | Clade1 |
| FR-D1 | France (Drôme) | F | Dogs | 1 | Clade1 |
| FR-D2 | France (Drôme) | F | Dogs | 1 | Clade1 |
| FR-D3 | France (Drôme) | F | Dogs | 1 | Clade1 |
| FR-D4 | France (Drôme) | F | Dogs | 1 | Clade1 |
| FR-D5 | France (Drôme) | F | Dogs | 1 | Clade1 |
| FR-D6 | France (Drôme) | F | Dogs | 1 | Clade1 |
| FR-D7 | France (Drôme) | M | Dogs | 1 | Clade1 |
| FR-G11 | France (Gard) | F | Environment | 3 | Clade1 |
| FR-G13 | France (Gard) | F | Environment | 3 | Clade1 |
| FR-G14 | France (Gard) | F | Environment | 1 | Clade1 |
| FR-G1 | France (Gard) | F | Dogs | 1 | Clade1 |
| FR-G2 | France (Gard) | F | Dogs | 1 | Clade1 |
| FR-G3 | France (Gard) | F | Dogs | 1 | Clade1 |
| FR-G4 | France (Gard) | F | Dogs | 3 | Clade1 |
| FR-G5 | France (Gard) | F | Dogs | 1 | Clade1 |
| FR-G6 | France (Gard) | N | Dogs | 1 | Clade1 |
| FR-G7 | France (Gard) | N | Dogs | 1 | Clade1 |
| FR-G9 | France (Gard) | M | Dogs | 1 | Clade1 |
| FR-V4 | France (Var) | F | Dogs | 3 | Clade1 |
| FR-V5 | France (Var) | F | Dogs | 3 | Clade1 |
| FR-V6 | France (Var) | F | Dogs | 1 | Clade1 |
| FR-V8 | France (Var) | M | Dogs | 1 | Clade1 |
| FR-V7 | France (Var) | M | Dogs | 1 | Clade1 |
| SEN-1 | Senegal (Dakar) | F | Dogs | 7 | Clade2 |
| SEN-3 | Senegal (Dakar) | F | Dogs | 7 | Clade2 |
| SEN-4 | Senegal (Dakar) | F | Dogs | 8 | Clade2 |
| SEN-5 | Senegal (Dakar) | F | Dogs | 7 | Clade2 |
| SEN-6 | Senegal (Dakar) | M | Dogs | 6 | Clade2 |
| SEN-7 | Senegal (Dakar) | M | Dogs | 8 | Clade2 |

**Additional file 1: Table S1.** Samples used for the 16s rDNA high throughput sequencing

**Additional file 1: Table S2.** Alpha diversity indices.

| **samples** | **sobs^1^** | **chao^2^** | **1/λ^3^** | ***H'*^4^** | **coverage** |
| --- | --- | --- | --- | --- | --- |
|  |  |  |  |  |  |
| AR-1 | 486 | 488,619048 | 9,200871 | 3,221985 | 0,999934 |
| AR-2 | 163 | 169,12 | 1,14684 | 0,430097 | 0,99992 |
| AR-3 | 179 | 180,666667 | 8,995829 | 3,263932 | 0,999951 |
| AR-4 | 384 | 386,142857 | 7,545972 | 3,347905 | 0,999963 |
| AR-5 | 552 | 561,893617 | 20,069243 | 3,893797 | 0,999833 |
| FR-CO1 | 388 | 397,893617 | 2,970822 | 1,974125 | 0,999775 |
| FR-CO2 | 163 | 170,125 | 1,024968 | 0,117396 | 0,999912 |
| FR-CO3 | 410 | 425,681818 | 2,185317 | 1,720119 | 0,999661 |
| FR-CO4 | 187 | 191,275 | 1,018813 | 0,09275 | 0,999928 |
| FR-CO7 | 375 | 378,461538 | 24,522167 | 4,054714 | 0,999921 |
| FR-D1 | 116 | 131,4 | 3,32099 | 1,588577 | 0,999878 |
| FR-D2 | 353 | 357,5 | 21,261018 | 3,966647 | 0,999936 |
| FR-D3 | 403 | 410,631579 | 1,33944 | 0,776058 | 0,999869 |
| FR-D4 | 502 | 508,487179 | 3,761862 | 2,533045 | 0,999869 |
| FR-D5 | 282 | 296,25 | 1,970625 | 1,52713 | 0,999852 |
| FR-D6 | 199 | 229,333333 | 6,075056 | 2,901061 | 0,999874 |
| FR-D7 | 16 | 34 | 1,729779 | 0,65445 | 0,999951 |
| FR-G1 | 301 | 312,756757 | 1,160085 | 0,538 | 0,999831 |
| FR-G2 | 207 | 229 | 12,416449 | 3,155439 | 0,999907 |
| FR-G3 | 412 | 425,046512 | 1,360248 | 0,943209 | 0,99981 |
| FR-G4 | 252 | 273,857143 | 2,779418 | 2,132844 | 0,999898 |
| FR-G6 | 232 | 241,545455 | 18,586129 | 3,414094 | 0,999824 |
| FR-G7 | 177 | 181,583333 | 1,126983 | 0,410272 | 0,999935 |
| FR-G13 | 178 | 188,2 | 1,366714 | 0,894955 | 0,999889 |
| FR-G14 | 202 | 217 | 3,212836 | 2,290781 | 0,999933 |
| FR-G5 | 213 | 285,526316 | 15,615973 | 3,335171 | 0,999589 |
| FR-G11 | 116 | 155,545455 | 1,01762 | 0,078972 | 0,999877 |
| FR-G9 | 47 | 152 | 1,661546 | 0,817595 | 0,999925 |
| FR-V4 | 171 | 176 | 2,18374 | 1,737319 | 0,999982 |
| FR-V5 | 106 | 148,5 | 1,080719 | 0,214623 | 0,999755 |
| FR-V6 | 289 | 301 | 5,258795 | 2,736407 | 0,99993 |
| FR-V7 | 55 | 61 | 1,004835 | 0,023517 | 0,999966 |
| FR-V8 | 229 | 229,75 | 15,88867 | 3,546918 | 0,999971 |
| SEN-1 | 135 | 142,5 | 1,792541 | 1,462579 | 0,999974 |
| SEN-3 | 78 | 84 | 1,014384 | 0,066575 | 0,999953 |
| SEN-4 | 65 | 66,5 | 1,093591 | 0,28999 | 0,999985 |
| SEN-5 | 125 | 127 | 1,471469 | 1,068987 | 0,999971 |
| SEN-6 | 109 | 114 | 6,491046 | 2,695312 | 0,999955 |
| SEN-7 | 138 | 139,666667 | 3,232085 | 1,925912 | 0,999966 |
| SEN-8 | 143 | 152,333333 | 1,34234 | 0,86708 | 0,999953 |

^1^Observed richness

^2^Chao1 richness index

^3^Inverse of Simpson diversity index

^4^Shannon diversity index
